# Complex Thoracic Aortic Diseases and Surgery: A Quest for the Golden Fleece

**DOI:** 10.3390/jcm13216620

**Published:** 2024-11-04

**Authors:** Dimitrios E. Magouliotis, Thanos Athanasiou

**Affiliations:** 1 Department of Cardiac Surgery Research, Lankenau Institute for Medical Research, Main Line Health, Wynnewood, PA 19096, USA; 2 Unit of Quality Improvement, Department of Cardiothoracic Surgery, University of Thessaly, Biopolis, 41110 Larissa, Greece; 3 Department of Surgery and Cancer, Imperial College London, St Mary’s Hospital, London W2 1NY, UK; t.athanasiou@imperial.ac.uk

In Greek mythology, the Golden Fleece, a golden-wooled fleece of a winged ram called Chrysomallos, stood as an eternal symbol of authority and power [1]. According to the legend, the hero Jason, accompanied by his crew of Argonauts, set sail to find and acquire the Golden Fleece in order to be rightfully placed on the throne of Iolcus, an ancient city in Thessaly, Greece. In recent times, the appropriate management of complex aortic diseases represents the modern Golden Fleece for every aortic surgeon, along with all the adjacent specialties. Starting from the point of understanding the biology and pathogenesis of aortic diseases [2,3] and moving to enhance our diagnostic and treatment armamentarium [4,5], we can proudly admit that we have made tremendous steps forward over the last decades in our quest for the Golden Fleece of the appropriate management of complex aortic diseases. In this context, we decided to edit this Special Issue aiming to highlight the latest advances in this field.

Early diagnosis represents an important pillar in the appropriate management of complex aortic diseases that enables a more tailor-made treatment approach, thus providing enhanced outcomes for each patient [6,7,8,9]. In this context, the discovery of novel biomarkers is crucial [10,11]. In the present Special Issue, the cardiac surgery group from the University of North Carolina demonstrated the implication of extracellular matrix metalloproteinase inducer (EMMPRIN) in Marfan syndrome-related Thoracic Aortic Aneurysm (TAA) biology [Contribution 1]. According to their findings, low EMMPRIN levels, in conjunction with other matrix metalloproteinases, distinguished Marfan syndrome-related TAAs from healthy controls, suggesting its potential role as a diagnostic biomarker [Contribution 1]. In fact, the importance of such biomarkers in the diagnostic process and treatment management was further investigated and summarized in the review article conducted by the same team and included in the current Special Issue [Contribution 2]. In that review [Contribution 2], the authors summarized the new opportunities brought by the rise in machine learning and artificial intelligence in preoperative imaging and computer-assisted aortic measurements, along with the biochemical monitoring using advanced genetic and genomics/proteomics methodologies. All these recent advances shed new light into the pathogenesis of TAAs and provide new opportunities for early diagnosis and enhanced treatment design and management.

The importance of accurate preoperative imaging was further highlighted in the article by Koulouroudias et al. [Contribution 3]. In that article [Contribution 3], the authors investigated the role of imaging during the preoperative planning for a frozen elephant trunk procedure and discussed the nuisances and uncertainties of sizing using three index cases: Type A aortic dissection, distal thoracic aortic aneurysm, and chronic dissection. On the other hand, Mylonas et al. [Contribution 4] evaluated the endovascular treatment approach for the management of Type A Aortic Dissection (TAAD). According to that meta-analysis, thoracic endovascular aortic repair (TEVAR) seems to be feasible in highly selected patients with TAAD [Contribution 4]. Nonetheless, overcoming technical limitations and acquiring long-term data represent the future goals of making this approach more accessible [Contribution 4]. Furthermore, Kudo et al. [Contribution 5] evaluated the effectiveness of Zone 1-Landing hybrid TEVAR by comparing its outcomes with those of Zone 2-Landing hybrid TEVAR. The authors concluded that Zone 1- and 2-landing hybrid TEVAR outcomes were similarly satisfactory [Contribution 5].

All these advances in the surgical management of complex aortic diseases represent optimistic signs for the new era that rises in aortic surgery—the era of less invasive and more individualized approaches for the treatment of aortic diseases [12,13,14,15]. According to the Greek myth, Jason, after many difficulties, managed to acquire the Golden Fleece, but the journey back to his city was not easy [1]. Even his ship, the famous Argo, was sunk. In the same way, we may surpass modern surgical limitations in the treatment of aortic diseases during the upcoming decades. Nonetheless, the implementation of novel techniques, technologies (e.g., artificial intelligence and custom devices), and biomarkers necessitates strong and valid evidence, along with constant quality improvement initiatives to provide optimal healthcare service in a safe and feasible manner.
